# Norwegian trauma team leaders - training and experience: A national point prevalence study

**DOI:** 10.1186/1757-7241-19-54

**Published:** 2011-10-05

**Authors:** Amund Hovengen Ringen, Magnus Hjortdahl, Torben Wisborg

**Affiliations:** 1 The BEST Foundation: Better & Systematic Team Training; Hammerfest Hospital, Department of Anaesthesiology and Intensive Care, Finnmark Health Trust, Hammerfest, Norway; 2 Anaesthesia and Critical Care Research Group, Faculty of Health Sciences, University of Tromsø, Norway

**Keywords:** Trauma team, leadership, training, non-technical skills, leader experience

## Abstract

**Background:**

The treatment of trauma victims is a complex multi-professional task in a stressful environment. We previously found that trauma team members perceive leadership as the most important human factor. The aim of the present study was to assess the experience and education of Norwegian trauma team leaders, and allow them to describe their perceived educational needs.

**Methods:**

We conducted an anonymous descriptive study using a point prevalence methodology based on written questionnaires. All 45 hospitals in Norway receiving severely injured trauma victims were contacted on a randomly selected weeknight during November 2009. Team leaders were asked to specify what trauma related training programs they had participated in, how much experience they had, and what further training they wished, if any.

**Results:**

Response rate was 82%. Slightly more than half of the team leaders were residents. The median working experience as a surgeon among team leaders was 7.5 years. Sixty-eight percent had participated in multi-professional training in non-technical skills, while 54% had passed the advanced trauma life support(ATLS) course. Fifty-one percent were trained in damage control surgery. A median of one course per team leader was needed to comply with the new proposed national standards. Team leaders considered training in damage control surgery the most needed educational objective.

**Conclusions:**

Level of experience among team leaders was highly variable and their educational background insufficient according to international and proposed national standards. Proposed national standards should be urgently implemented to ensure equal access to high quality trauma care.

## Background

Trauma is the leading cause of death among individuals younger than 35 years of age in Norway [1]. Several studies indicate that some of these deaths can be prevented [2-5]. The treatment of trauma victims is a complex, multi-professional task in a stressful environment, and patient outcome is dependent on correct decisions and priorities undertaken at the right time. Trauma teams, which are specialised groups of doctors, nurses, and other personnel aimed at improving trauma care, were introduced in the early 1970s [6]. Teamwork now plays an important role in assuring patient safety [7]. Although trauma team composition varies, the trauma team is invariably led by a team leader, and in most Norwegian hospitals the team leader is a surgeon.

In a previous study, we found that leadership was perceived as the most important human factor by trauma team members [8]. In this qualitative study, team members and leaders revealed that the ideal leader should be an experienced surgeon, have extensive knowledge of trauma care, communicate clearly, and radiate confidence. We also found that the team considered experience a key prerequisite for functional leadership.However, we were surprised to find that several of the team leaders interviewed were inexperienced and had little knowledge of trauma care. Team leaders stated that more experience and better training are important to them in order to become better leaders.

Norway has 45 hospitals designated to receive severely injured patients [9]. This is due to geography, demography, and politics, despite a population of less than 5 million inhabitants. The hospitals vary from minor community hospitals to university hospitals. While a well-developed air ambulance system is available, weather and logistics regularly prohibit or delay patient transfer [10]. University hospitals function as regional trauma centres, but all of the 45 hospitals are expected to perform initial trauma care [11]. All hospitals receiving severely injured trauma patients have predefined trauma teams [12,13] and have surgeons with specialist accreditation on call, although they may be on call from their home.

There are no national requirements for trauma team composition in Norway and standards for team leaders do not exist [14]. This may partly explain the variability in experience level among trauma team leaders. Surgeons may have team leader obligations in a hospital for several years without gaining a significant amount of experience treating trauma patients. Even in areas with high population density, the amount of experience among doctors in the trauma team will vary. One study showed that even at larger trauma centres in the United States, residents do not get enough experience in operative treatment of trauma patients [15].

Guidelines for trauma care in Norway have been proposed but not yet implemented in all regions, as this is dependent on local political processes. A national working group developed standards for hospitals intending to receive and treat victims of major injury in 2007, and provided specified requirements for trauma team leaders [14]. Although the proposal requires different skills and training at different hospital levels, the expectations of the team leader (and the anaesthesiologist) are similar. They should both be certified in Advanced Trauma Life Support (ATLS). All team members should preferably have similar skills training, appropriate to their profession. The team leader or his consultant on call should be trained in emergency haemostatic surgery. All team members should regularly participate in team training focusing on non-technical skills, such as communication, cooperation, leadership, and decision-making. These requirements are comparable to those defined by the American College of Surgeons Committee on Trauma in the "Resources for the optimal care of the injured patient" [16].

There seems to be a mismatch between expectations of team leaders from team members and a reported lack of skills and knowledge from some team leaders. As this was a surprising finding in the previous qualitative study, which was not aimed to be representative, we saw a need for a better description of the present status concerning trauma team leaders in Norway as a starting point for improvement. The newly proposed standards for Norway made a natural reference for comparison between the present state and what would be desirable. The aim of the present study was to assess the experience and education of Norwegian trauma team leaders, and allow them to describe their perceived educational needs.

## Methods

### Study design

We conducted a descriptive study using a point prevalence methodology based on written questionnaires.

### Data collection and sampling

We contacted all 45 hospitals in Norway receiving severely injured trauma victims on a randomly selected week night during November 2009. We asked the coordinator in the emergency department (ED) for the name of the trauma team leader on call on that specific night. In the following weeks, we mailed all team leaders a questionnaire. Not all EDs were reached on the first attempt, and a follow-up call was performed on a similar week night eight weeks later.

The questionnaire consisted of the following items:

#### Professional experience

Team leaders were characterised as certified specialists or still in training. Their present specialty was asked for, as was length of experience in that specialty and as a trauma team leader.

#### Education

Team leaders were asked to specify which trauma-related training programs they had participated in. We focused on three different types of training programs:

1. Training in skills concerning the initial examination and treatment of trauma patients (ATLS).

2. Training in emergency haemostatic surgery/damage control surgery.

3. Multi-professional team training in non-technical skills, such as communication, cooperation, leadership, and decision-making.

### Approval

The Norwegian Social Science Data Services (ref. 22098) approved the study. The Regional Committee for Medical Research (Health Region North) did not consider any need for approval, given the nature of the study (2009/106-14). The questionnaire was anonymous, and no answers could be traced to individual respondents or hospitals.

## Results

Responses were received from 37 of 45 possible team leaders at the 45 hospitals receiving severely injured patients at the time of the study, for a response rate of 82%. Twenty of the 37 teams (54%) were led by residents. Surgeons or surgical residents were trauma team leaders in 31 of 37 hospitals (84%); of the remaining team leaders, three were orthopaedists, two were surgeons and orthopaedists (double specialty), and one was an anaesthesiologist. Team leaders had a median of 7.5 years of experience in their specialty, (interquartile range (IQR) 4 - 19). Four team leaders had less than one year of experience in their specialty. Seven teams were led by team leaders with less than one year of experience as a trauma team leader.

Team leaders were asked whether they felt sufficiently experienced to act as a team leader. Five of 36 (14%) responding team leaders reported insufficient experience to undertake the obligation as a team leader. When asked about training, 17 of 34 respondents (50%) answered that they felt they had sufficient training, while the other half felt a need for further courses to act as a team leader.

**Figure 1:**
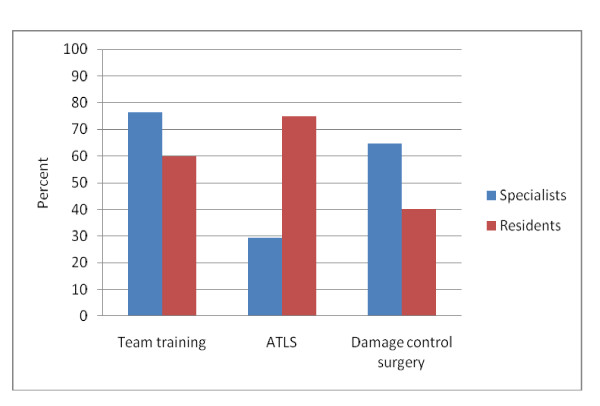
**Present training of Norwegian trauma team leaders**. The bars represents residents and specialists in the survey fulfilling proposed national requirements concerning courses in non-technical, multi-professional team skills (team training); advanced trauma life support course (ATLS); and damage control surgery as per November 2009

Sixty-eight percent of the team leaders had participated in multi-professional team training courses focusing on non-technical skills, such as communication, cooperation, leadership, and decision-making. Fifty-four percent had passed the ATLS course and 51% were trained in damage control surgery (Figure 1).

We asked the participants which training program they considered most needed to improve their trauma readiness. Ten out of 26 answered that training in haemostatic damage control surgery were most needed, while ATLS course participation was rated second most important. Several informants pointed out the need for minimal standards and regular training as important for trauma readiness.

Seven of 37 team leaders (19%) fulfilled the proposed Norwegian trauma system standards concerning individual skills as described in the Introduction. In total, the 37 team leaders were lacking 46 courses to reach the recommended level. We found a need for a median of 1 course (IQR 1-2) per team leader.

## Discussion

This study found great variability in experience level among Norwegian trauma team leaders. Due to geography and demographics, it is likely that team leaders in some Norwegian hospitals seldom treat severely injured patients. Lack of everyday exposure to these patients makes the need for training much more important. We found that many trauma team leaders have had several courses in different aspects of trauma care. However, the complexity of trauma treatment depends on a leader with knowledge of leadership and teamwork, as well as principles of examination and prioritization [7]. This background is incorporated into the proposed national standards that define three different courses: haemostatic damage control trauma surgery, teamwork, and non-technical skills training in teams. In our group of respondents, only a minority fulfilled these standards.

In a previous study, we found that trauma knowledge, experience, and training were perceived as key factors of good leadership [8], and Hansen et al. found that team leaders have a subjective improvement in trauma skills after training in haemostatic surgery [17]. A Danish study indicated that inexperienced team leaders lack the ability to delegate tasks to other team members [18]. In Norwegian trauma teams, the leader might be one of the least experienced members of the team. Høyer et al. [18] suggest emphasizing leadership and communication in the education of junior residents, which might provide inexperienced team leaders with the ability to lead the team while also making use of more experienced team members. There are good reasons to believe that trauma team leaders with only short practical experience and little training in trauma surgery and team work are unlikely to perform optimally [19]. This might lead to negative consequences not only for the team leader and members, but may also affect patient outcomes.

There are no national requirements for training of Norwegian trauma team leaders, and the proposed national standard [14] is a natural starting point to assess educational needs. To educate this group of 37 team leaders to the recommended level, 46 courses were needed. If the average surgeon on call completes one night shift a week, we have stipulated a need of 392 course participants to bring all team leaders in all Norwegian hospitals up to the proposed acceptable standard. A great majority of the residents have passed ATLS training but need more training in damage control surgery. We claim that this is a manageable challenge, and it is therefore realistic to introduce the proposed standards to Norwegian hospitals. Even when the leaders have completed the three different courses, further education will be required. To maintain readiness, the proposed national standard suggests that courses in damage control surgery be repeated every third to fifth year, and that regular team training in non-technical skills be repeated on a regular basis, no more than three months after appointment of new team members. Several respondents mentioned repetition and frequent training as key factors.

This study has several limitations. First, this is an observational study and our data are based on two randomly selected days. Although the data does not necessarily depict all Norwegian trauma leaders, there is no reason to believe that data obtained on other nights would be significantly different. One could argue that it is common for only junior staff to be on call during night shifts, and therefore conducting a survey during the night may have biased the results toward less experienced team leaders. On the other hand, conducting the same survey during regular work hours might provide a false sense of the level of experience and training of trauma team leaders. Because the study is based on anonymous questionnaires, there is always a possibility of under- or over-reporting of personal skills; however, the anonymous nature and the public acknowledgement that skills are lacking should reduce this bias.

In conclusion, the present state of Norwegian trauma team leader training is clearly insufficient when compared to international criteria such as the American College of Surgeons - Committee on Trauma requirements [16] or the proposed national Norwegian standard [14].There is a need for intensified training of trauma team leaders; however, the amount of course seats needed is achievable.

## Competing interests

The author declares that they have no competing interests.

## Authors' contributions

TW conceived the study. AHR, MH, and TW designed the study. AHR, MH, and TW reviewed the literature. AHR and MH collected the data. AHR, MH, and TW wrote the manuscript. All the authors revised and approved the manuscript.

## References

[B1] Statistics Norway Deaths, by sex, age and underlying cause of death. The whole country. 2009 http://www.ssb.no/english/subjects/03/01/10/dodsarsak_en/tab-2010-12-03-02-en.html (accessed 24th March 2011)

[B2] Esposito TJ Sanddal ND Hansen JD Reynolds S Analysis of preventable trauma deaths and inappropriate trauma care in rural state J Trauma 1995 39 955 962 10.1097/00005373-199511000-00022 7474014

[B3] Esposito T Sanddal T Reynolds S Sanddal N Effect of a voluntary trauma system on preventable death and inappropriate care in a rural state J Trauma 2003 54 663 9 10.1097/01.TA.0000058124.78958.6B 12707527

[B4] Chiara O Scott JD Cimbanassi S Marini A Zoia R Rodriguez A Scalea T Milan Trauma Care Study Group Trauma deaths in an Italian urban area: an audit of pre-hospital and in-hospital trauma care Injury 2002 33 553 562 10.1016/S0020-1383(02)00123-7 12208056

[B5] Chua WC D'Amours SK Sugrue M Caldwell E Brown K Performance and consistency of care in admitted trauma patients: our next great opportunity in trauma care? ANZ J Surg 2009 79 443 8 10.1111/j.1445-2197.2009.04946.x 19566867

[B6] Brooks A Burton B Williams J Mahoney P Trauma teams Trauma 2001 3 211 5

[B7] Manser T Teamwork and patient safety in dynamic domains of healtcare: a review of the literature ActaAnaesthesiologica Scandinavia 2009 53 143 151 10.1111/j.1399-6576.2008.01717.x 19032571

[B8] Hjortdahl M Ringen AH Naess AC Wisborg T Leadership is the essential nontechnical skill in the trauma team-results of a qualitative study Scand J Trauma ResuscEmerg Med 2009 17 48 10.1186/1757-7241-17-48 PMC2764560 19781093

[B9] Kristiansen T Lossius HM Søreide K Steen PA Gaarder C Næss PA Patients Referred to a Norwegian Trauma Centre: effect of transfer distance on injury patterns, use of resources and outcomes J Trauma Manag Outcomes 2011 16 5 9 10.1186/1752-2897-5-9 PMC3135518 21679393

[B10] Haug B Avall A Monsen SA Reliability of air ambulances--a survey in three municipalities in Helgeland Tidsskr Nor Laegeforen 2009 129 Norwegian 1089 93 19488089 10.4045/tidsskr.08.0306

[B11] Kristiansen T Søreide K Ringdal KG Rehn M Krüger AJ Reite A Meling T Naess PA Lossius HM Trauma systems and early management of severe injuries in Scandinavia: review of the current state Injury 2010 41 444 52 10.1016/j.injury.2009.05.027 19540486

[B12] Isaksen MI Wisborg T Brattebø G Organisation of trauma services--major improvements over four years Tidsskr Nor Laegeforen 2006 126 Norwegian 145 7 16415933

[B13] Larsen KT Uleberg O Skogvoll E Differences in trauma team activation criteria among Norwegian hospitals Scand J Trauma ResuscEmerg Med 2010 18 21 10.1186/1757-7241-18-21 PMC2874509 20406456

[B14] National working group, Report on organization on treatment of seriously injured patients - Trauma system. [Organisering av behandlingen av alvorlig skadde pasienter - Traumesystem]. In Norwegian. Oslo, South-East Regional Health Trust, 2007 http://old.helse-sorost.no/stream_file.asp?iEntityId=1567 (accessed 24th March 2011)

[B15] Fakhry SM Watts DD Michetti C Hunt JP The Resident Experience on Trauma: Declining Surgical Opportunities and Career Incentives? Analysis of Data from a Large Multi-institutional Study J Trauma 2003 54 1 8 10.1097/00005373-200301000-00001 12544893

[B16] American college of surgeons, Committee on Trauma. Resources for the optimal care of the injured patient. 2006. Chicago 2006 American College of Surgeons

[B17] Hansen K Uggen PE Brattebø G Wisborg T Training operating room teams in damage control surgery for trauma: A followup study of the Norwegian model J Am CollSurg 2007 205 712 716 10.1016/j.jamcollsurg.2007.06.015 17964448

[B18] Høyer C B Christensen EF Eika B Junior physician skill and behaviour in resuscitation: A simulation study Resuscitation 2009 80 244 248 10.1016/j.resuscitation.2008.10.029 19084318

[B19] Cooper S Wakelam A Leadership of resuscitation teams: ''Lighthouse Leadership'' Resuscitation 1999 42 27 45 10.1016/S0300-9572(99)00080-5 10524729

